# When are researchers willing to share their data? – Impacts of values and uncertainty on open data in academia

**DOI:** 10.1371/journal.pone.0234172

**Published:** 2020-07-01

**Authors:** Stefan Stieglitz, Konstantin Wilms, Milad Mirbabaie, Lennart Hofeditz, Bela Brenger, Ania López, Stephanie Rehwald

**Affiliations:** 1 University of Duisburg-Essen, Essen, Germany; 2 University of Bremen, Bremen, Germany; 3 RWTH Aachen, Aachen, Germany; Universitat de Valencia, SPAIN

## Abstract

**Background:**

E-science technologies have significantly increased the availability of data. Research grant providers such as the European Union increasingly require open access publishing of research results and data. However, despite its significance to research, the adoption rate of open data technology remains low across all disciplines, especially in Europe where research has primarily focused on technical solutions (such as Zenodo or the Open Science Framework) or considered only parts of the issue.

**Methods and findings:**

In this study, we emphasized the non-technical factors perceived value and uncertainty factors in the context of academia, which impact researchers’ acceptance of open data–the idea that researchers should not only publish their findings in the form of articles or reports, but also share the corresponding raw data sets. We present the results of a broad quantitative analysis including N = 995 researchers from 13 large to medium-sized universities in Germany. In order to test 11 hypotheses regarding researchers’ intentions to share their data, as well as detect any hierarchical or disciplinary differences, we employed a structured equation model (SEM) following the partial least squares (PLS) modeling approach.

**Conclusions:**

Grounded in the value-based theory, this article proclaims that most individuals in academia embrace open data when the perceived advantages outweigh the disadvantages. Furthermore, uncertainty factors impact the perceived value (consisting of the perceived advantages and disadvantages) of sharing research data. We found that researchers’ assumptions about effort required during the data preparation process were diminished by awareness of e-science technologies (such as Zenodo or the Open Science Framework), which also increased their tendency to perceive personal benefits via data exchange. Uncertainty factors seem to influence the intention to share data. Effects differ between disciplines and hierarchical levels.

## Introduction

With the advance of digitization, the supply of data available to researchers has continued to grow. Accurate research data management (RDM) has therefore become increasingly important. Along with the proliferation of data-intensive research processes (e.g. online experiments), researchers and funding agencies have started to call on data intensive research disciplines to provide open data exchange [1].

Open data is the idea that researchers and practitioners–such as governments and other institutions–should not only publish their findings in the form of articles or reports, but also share the corresponding raw data sets. Endorsing this idea may be economically motivated–in order to provide benefits for developers, citizens or companies–as well as socially motivated–in order to provide transparency, accountability, responsiveness, and participation [2]. Open data is one important part of the open science phenomenon, which encourages openness and connectivity with respect to research data [3]. In contrast to other principles of open science, open data has been insufficiently explored–particularly in European academia. In this article, the term open data is therefore considered exclusively from an academic perspective.

Various universities and third-party online platforms have started to develop diverse e-science technologies (e.g. data repositories and digital research environments) to allow researchers to share their resources and raw data sets without restrictions [4]. Some noteworthy examples of e-science technologies are the open data repository Zenodo [5], the university cloud service Sciebo [6], and the Open Science Framework [7]. Although several studies have promoted the benefits of open data, the latest research demonstrates a low willingness to share data across those platforms [8,9]. Accordingly, scientific strategies are needed in order to efficiently promote open data [10].

Although much research deals with the implementation and adoption of open data practices in medical and governmental contexts, it has been largely overlooked in academia–particularly in the European research context. This conflicts with the demands of institutions and funding agencies such as the European Union, which promote open access to research data [11]. Considering the infrastructures and needs of European universities and research institutions, research has primarily examined researchers’ technical requirements and expectations towards technology [8]. While technical barriers represent one of the two pillars of open data acceptance, non-technical barriers (such as the perceived advantages and disadvantages of sharing data) have been mostly neglected [12]. Since European academia differs from, for example, US academia in its understanding of data protection and privacy–as well as in its institutional structures–a more detailed examination of the value of sharing research data in Europe is needed. Although some studies have considered individual, non-technical barriers [13–15], research regarding European academia is limited, and it has rarely looked at how these factors covary in different disciplines. Although previous research has indicated that different barriers between research disciplines exist [16,17], knowledge of these differences–especially in European academia–is very limited. The influence of hierarchical differences in the willingness to share data is also largely unexplored. An overview of all factors that determine when European researchers share their data has not yet been achieved in any study. Non-technical barriers exist due to ethical or legal restrictions, as well as individual factors inhibiting researchers from sharing data [12]. Individual factors are represented by positive and negative drivers that directly influence an individual’s attitudes towards open data [4]. Besides positive and negative determinants, uncertainty factors appear to directly influence researchers’ intentions to share research data [18]. In view of the current discussion on open data, especially in Europe, it is essential to examine whether researchers’ refusals are caused by uncertainties or by a perceived low value of making research data publicly available.

In the literature on data sharing there is only rudimentary knowledge of individual uncertainty factors regarding open data in European academia. A multilevel research model based on inference statistical research was proclaimed to examine the impact of individual factors on researchers’ data sharing behavior and the cross-level interplay of uncertainty factors [12,18,19]. Therefore, this article introduces a novel research model based on the value-based theory [20]and prospect theory [21] to examine researchers’ individual factors and uncertainty factors. While the value-based theory assumes that an individual decides against a new alternative so long as the alternative’s disadvantages outweigh its perceived advantages, the prospect theory [21] assumes that individual uncertainties influence an individual’s decision-making. Thus, we examined the following research question:

*How do uncertainty factors and perceived value impact researchers’ intentions to adopt the open data process (e*.*g*. *publishing*, *reusing)*?

The aim of this article is to enrich the current debate on e-science technology implementation. Knowledge of how specific drivers influence researchers’ decision-making processes is of great significance for the development of internal implementation strategies [1,22]. Therefore, our research enhances the recognition of researchers’ difficulties with the implementation phase of open data practices, as well as offering European universities and research facilities potential strategies for addressing these difficulties. The results and insights of this research paper are highly relevant for Information Systems (IS) research since RDM, open access, and data reusability in science constitute a relevant, ongoing topic [1,10,12].

This work is structured as follows: First, we define RDM and open data. Next, we explain both theories mentioned above within the context of open data. Then, we explain and present the presumed impact of individual factors, including uncertainty factors and derived hypotheses. We present a novel research model, as well as the results of the largest open data survey in Germany to date. Finally, we discuss the analyzed research data and derived research implications.

## Definition of research data management

The overall aim of RDM is to provide researchers with innovative, diverse knowledge discoveries and broad knowledge use and reuse [23]. Research disciplines use different types of data, starting from different aggregation types of data (raw data, processed data, etc.) with different requirements for storage, usage, and interpretation [9]. Even data and information which are needed to interpret the data (such as software code) are considered to be research data. The requirements for handling heterogeneous types of data can differ between disciplines [17]. RDM covers a lot of different requirements and also includes the management of data through an infrastructure–a means of long-term storage, data security, and open access, as well as communication between researchers from different disciplines [24]. Research Data Canada defines RDM as the storage, access, and receipt of data generated within an investigation [25]. A loss of data, which has occurred in the past [26,27], should not happen in the future, as these data can be the basis for current research [28]. Nevertheless, Vines et al. [27] have stated that, as a publication ages, the availability of its data decreases; that is, the probability that the data are available decreases by 17 percent annually from the release date.

One option is to increase the availability of data by making it open according to the principles of FAIR data (Findable, Accessible, Interoperable, and Reusable)–especially making it persistently available [29]. Within the open science phenomenon, open data is one of the most important components of RDM [7]. The National Science Foundation defines open data as “publicly available data structured in a way to be fully accessible and usable” [30]. Urgency to adopt open data practices has increased since several international research funding institutions, such as the National Science Foundation, the Australian Research Council, the European Union [11], and the German Research Foundation [31] have made research data accessibility a top priority [32].

While the call for open data practices in academia is not novel, recent studies indicate that adoption of open data practices has declined among diverse research communities [4,18]. Cases have been documented in which research data were withheld or lost, without the possibility of replication or reuse [33]. To protect publicly funded research from any allegation of misconduct [34] and to guarantee open access of data for the public, governments and funding agencies have started to call on research disciplines to embrace open data practices [1,9]. Of late, numerous international research funding institutions have set up open data policies, where compliance constitutes a prerequisite for funding [12].

RDM and open data come with a number of advantages for researchers. For instance, interdisciplinary cooperation of researchers [35] and co-authorship [19] can be increased. In addition, open data policies can increase citations and improve researchers’ reputations [36]. Indeed, researchers who provide access to their research data are quoted more often than those who do not [37]. Furthermore, open access to research data and results presents an opportunity to confirm or refute those results [38]. Accordingly, it offers a control function, which ultimately increases quality of research. According to the long tail theory, research data have the potential to generate further insights in other contexts, as long as the data are made available to research groups from other disciplines [39]. In addition, RDM can help overcome so-called bottleneck effects, as other research disciplines can also achieve more accurate results with more research data [40].

Although data sharing offers many opportunities and can accelerate the research process, it is still not a common practice [41], and data are more likely to be held back by researchers and scientific journals [42]. Citations are generally seen as a form of reward for the quoted researcher; however, sharing data is not based on this formalism and does not provide researchers with any perceptible recognition [43]. Therefore, sharing data has not been established within the research community [36]. In addition to a perceived lack of recognition, the fear that other researchers will publish the data first could be another reason for not sharing research data [44]. Negative criticism can be another possible explanation [45]. Many researchers also seem to fear misinterpretation or misuse of their data [46]. According to the Research Information Network [44] there is a possible additional expenditure for administration and increased perceived competitive pressure [18].

There is plenty of mistrust across several academic fields when it comes to recording, preserving, and sharing research data [9,14]. According to Tenopir et al. [36], researchers have inherent physical and psychological barriers that prevent good RDM from being deeply rooted in them. This is well documented in a study by Savage and Vickers [33], who found that only one in ten researchers would be willing to share their data with unknown scientists, despite data being requested from open access journals.

## Theoretical background

Non-technical barriers constitute another reason for the rejection of open data [12,19]. Non-technical barriers directly determine individuals’ value processing, which constitutes a significant aspect of the value-based theory [20]. The value-based theory is grounded on the general assumption that an individual’s decision-making process is based on a cost-benefit principle; that is, a rational agent–*homo economicus*–always acts to maximize utility and profit. Hence, within a decision-making process, a rational agent considers whether the advantages of an alternative outweigh its disadvantages. The trade-off between perceived advantages and disadvantages is exemplified in the concept of value [47]: value represents an overall estimation of an alternative on which the individual chooses its future behavior.

Classic acceptance models such as the well-known theory acceptance model (TAM) were developed to explain the adoption of traditional technologies that have mainly been used for work purposes and where adoption was mandatory [48]. That is why Kim et al. [20] designed their value-based model of technology acceptance, which includes multiple roles for individuals. There is little research that considers the perceived individual value of a concept such as open data. For example, Kim & Zhang [16] consider an attitude rather than a possible change of attitude based on a value. They used the theory of planned behavior and the theory of reasoned action in their research model to describe how beliefs influence behavioral intentions [49]. Kim et al. [16] considered these theories in combination with findings from prior research on data sharing, knowledge sharing, and technology adoption and emphasized three main attitudinal beliefs: perceived career benefits, perceived career risks, and perceived effort involved in data sharing [16]. The theory of planned behavior was represented by the normative influence, which influences the attitude towards data sharing and data sharing behavior. They concluded that minimizing risk and effort, as well as emphasizing the benefits and the development of positive norms, would have a positive impact on the willingness of STEM (science, technology, engineering, and mathematics) researchers to share their data [16]. However, Kim & Zhang [16] failed to consider that users of open data play a dual role: they are both technology users and service consumers [20]. When examining open data, a consumer point of view is needed as well. In the value-based model of Kim et al. [20] this is represented by the theory of consumer choice and decision-making. In contrast to value, attitude (a basic component of TPB) represents a “psychological tendency that is expressed by evaluating a particular entity with some degree of favor or disfavor” [20]. Attitude was shown to lead to a weak mediation of beliefs on adoption intention with several studies discussing their relationship [20]. Kim et al. [20] framed this less as an attitude and more as a perceived value regarding the use of a technology. As indicated by Kim et al. [20], the value-based theory can be successfully adapted to the research field of user acceptance and service adoption.

While the valuing process is described as a rational process, the existence of uncertainty factors is not considered. Therefore, the prospect theory by Kahneman and Tversky [21] is used within this work to explain the effect of expected uncertainty factors during an evaluation process. The prospect theory considers cognitive distortions to be significant factors within decision-making processes: individuals tend to be risk-averse with respect to some new alternative when it is associated with uncertainty [21]. Individuals reliably become anxious when facing situations with unknown outcomes. According to the prospect theory, individuals tend to prefer options with marginal but certain benefits over options with great but uncertain benefits; also, individuals tend to prefer options with higher risk over options with certain small losses as long as the higher risk option can potentially avoid losses altogether.

The research of Kim & Zhang [16] indicates that attitudinal beliefs such as perceived career benefits, risk, and perceived effort have a significant influence on STEM researchers’ willingness to share their data. This data is consistent with the findings of Kankanhalli et al. [50], Kim [20] and Kling & Spector [51], who pointed out the importance of academic recognition and reputation. It supports the results of Campbell et al. [52], Tenopir et al. [36] and Vickers [33], who examined perceived risks that increase the disposition of researchers to share data. The influence of perceived gains also coincides with the results of other studies [52,53]. These constructs are therefore a good basis on which to test the acceptance of sharing research data. Whereas Kim & Zhang [16] examined the attitude against open data without considering the consumer perspective of individuals, this article considers perceived value according to the value-based adoption model of technology [20]. Moreover, neither Kim et al. [20] nor Kim & Zhang [16] included the influence of uncertainty factors on intentions to share data in their research. Further theories, such as the prospect theory, could be used to identify these missing determinants. In this study, we used the prospect theory and the value-based model of technology acceptance [20] and the constructs of Kim & Zhang [16] in order to derive a new research model.

## Hypotheses and model development

Drawing on a confluence of the value-based theory [20] and the prospect theory [21], we developed a model to measure the impact of value and uncertainty factors on researchers’ compliance intention. According to the research presented above, perceived benefits and losses associated with a behavior–such as sharing research data–impact the perceived value of the behavior [20]. This value has an impact on researchers’ willingness to share their data and could be mediated by some uncertainty factors [21]. For each factor, we derived hypotheses based on the existing research [20,52,53].

### Perceived advantages (Hypotheses 1a-c)

Perceived advantages affect an individual’s perceived utility of switching to a new alternative and thus are seen as major reasons for changing attitudes [54]. Here performance advantages refer to perceived advantages researchers might enjoy by adopting open data practices, such as performance enhancement and quality enhancement [19]. Besides performance incentives, open data offers diverse personal advantages, for example increased citation rates, recognition, and reputation [14,19]. These aspects affect not only researchers’ impact factors [55], but also the relevance of their own publications, leading to increased peer recognition [56], new collaboration possibilities [57], and overall career benefits [16]. Thus, we generated the following hypotheses:

*H1a*: *Perceived switching advantages have a positive effect on researchers’ perceived value of open data*.

*H1b*: *Perceived career advantages have a positive effect on researchers’ perceived value of open data*.

*H1c*: *Perceived network possibilities have a positive effect on researchers’ perceived value of open data*.

### Perceived disadvantages (Hypotheses 2a-b)

The impact of disadvantageous factors on value evaluation has already been examined in various IS research investigations [19,58]. As the literature shows, non-monetary costs incurred by some change usually include time costs, effort costs, and psychological costs such as frustration and discomfort [59]. Performance disadvantages, here, refer to appraisals of how much additional work and expense is associated with open data. Individuals prefer to justify remaining in their current IT environments instead of making an effort to learn a new one [60]. Career disadvantages, here, refer to “a researcher's belief about the possibility of receiving an undesirable consequence on their careers from data sharing” [16], such as a loss of publication opportunities [33]. As indicated by Kim and Zhang [16], career risks influence researchers’ data sharing behavior to a great extent. Thus,

*H2a*: *Perceived switching disadvantages have a negative effect on researchers’ perceived value of open data*.

*H2b*: *Perceived career disadvantages have a negative effect on researchers’ perceived value of open data*.

### Perceived value (Hypothesis 3)

Value is here defined as a trans-situational goal varying in importance and serving as a guiding principle in an individual’s life [61]. Therefore, value is defined according to perceived gains and losses relative to a natural reference point [21], and it directly influences individuals’ behavior and decision-making [62]. Every time individuals evaluate the perceived value of some change as low, they develop a greater resistance towards the change [54]. In case of high valuation, individuals are less likely to resist the changes [63]. The positive and significant influence of perceived value on the adoption decision has already been demonstrated in previous research [20]. Thus,

*H3*: *Perceived value has a positive effect on researchers’ intentions to adopt open data practices*.

### Uncertainty factors (Hypotheses 4a-c)

Uncertain situations provoke anxiety and fear [48]. Uncertainty factors are defined as emotional psychological determinants “comprising worry regarding a potential threat as yet unidentified or unrealized accompanied by a similar—but attenuated—version of the physiological reaction to fear” [64]. In contrast to perceived advantages and disadvantages, uncertainty factors represent emotionally-based concerns about future injustice, and do not influence an action’s value [65,66]. For example, although many travelers are aware that flying is efficient and cost-effective, and that the risk of an accident remains low compared to other options, they might be frightened of flying and refuse to fly at all. This fear does not reflect a disadvantage of commercial air travel but rather emotionally-based concerns about the future deeply rooted in the personalities of individuals.

The same phenomenon applies to open data: despite the advantages clearly outweighing the disadvantages, uncertainty prevents researchers from embracing it. Therefore, uncertainty negatively influences usage behavior [67], which means individuals tend to maintain the status quo [68]. From researchers’ perspectives, uncertainty factors manifest in different aspects of open data. For instance, they fear that data sharing will result in increased competition and publication pressure [69]. In addition, researchers are concerned that they will not be the first to publish their own data if it is openly available [70], resulting in a fear of being replaced by others having access to the exclusive knowledge of one’s research data [71]. Fear of data misuse also plays a significant role: researchers stated that unawareness about the further use of their data, as well as a lack of control over it, prevents them from sharing [72]. An interview study by Enke et al. [73] indicated that fear of loss of control is the most relevant factor in withholding data. Thus,

*H4a*: *Fear of losing one’s unique value has a negative effect on researchers’ intentions to adopt open data practices*.

*H4b*: *Fear of data misuse has a negative effect on researchers’ intentions to adopt open data practices*.

*H4c*: *Fear of competition has a negative effect on researchers’ intentions to adopt open data practices*.

#### Additional assumptions

In this study, we also examined how disciplines differ in researchers’ willingness to share research data. Previous studies have indicated differences between some disciplines; however, a detailed overview of this subject is lacking. Kim & Zhang [16] considered STEM researchers’ attitudes against open data and Witt et al. [40] stated that there are further disciplines that could benefit from better RDM. Several studies have stated that there are differences in perceived advantages and disadvantages regarding RDM between disciplines [74,75]. It can be assumed that these differences also concern open data. A few studies have provided more detailed insights. Wessels et al. [76] concluded that willingness to share research data depends on where these data are shared and with whom. According to Akers and Doty [17], just one third of social scientists are willing to share their data outside the research group. Overall, humanities scholars seem to be less willing to share their data than other disciplines [18]. This suggests that disciplines differ in researchers’ willingness to conduct research that implements research data sharing and reuse practices (open data). Thus,

*H5*: *Researchers differ according to their disciplines in their perceptions of the value of open data*.

Kim & Zhang [16] collected data on the relation between academic roles and intentions to adopt open data practices, but they did not consider hierarchical factors. Previous research has pointed out that fear of a loss of power influences the willingness to apply knowledge management in commercial organizations [77,78]. Knowledge management concerns the management of data, information, and all findings that are useful for organization [79], which overlaps with many aspects of RDM and open data. Power structures and hierarchical levels can also influence researchers’ willingness to share data within academic institutions. Due to the differences in power, it can be assumed that professors differ from PhD candidates and other academic staff, for instance, in their perceived value of open data. Thus,

*H6*: *Researchers differ in their perceived value of open data according to their hierarchical levels*.

In order to test our hypotheses, we derived a research model, which we analyzed with a structural equation model following the established PLS approach [80,81]. We also surveyed researchers (N = 995) from different disciplines about their attitudes toward open data.

## Methodology

To validate our research model and to identify the factors that support or hinder open data practices in academia, we designed and conducted a large-scale study using an online questionnaire. We collected accounts of researchers’ opinions on RDM practices and requirements, and tested our research model. The study focused on researchers at German universities. Nine large universities (more than 20,000 students), and four medium-sized universities (more than 10,000 students) agreed to run the survey among their researchers. The survey proceeded between March 2018 and January 2019. To maintain participants’ anonymity, we did not collect data on age or gender. Participation was voluntary, and the questionnaire was in English to avoid translation bias. The aim of this data evaluation was to identity key constructs that influence the intention to share research data, rather than to carry out theory testing, theory confirmation, or the comparison of alternative theories.

### Item development

We adapted most of the constructs from existing scales (see Table 1). Since we could not identify adequate scales for the factors “network possibilities”, “fear of competition”, and “fear of misuse”, we developed new scales. First, during a group discussion including six researchers from different scientific disciplines (IS, physics, mathematics, computer science, psychology, and economics), we designed items for these constructs. Second, we conducted an open sorting process [82] to assure items’ validity. For this step, we conducted knowledge from70 researchers from different German universities. The 70 participants were from diverse scientific areas (30% social sciences, 35% engineering, 7% life sciences, and 28% nature sciences) and were briefed about RDM and open data in a workshop. Later, we rejected all items assigned by less than 61% of the participants [83].

**Table 1:** Constructs tested in the survey.

Construct	Short	Source
Intention to Share Research Data	IS	[88]
Perceived Value	PV	[54]
Switching Advantages	SA	[54]
Career Advantages	CA	[16]
Network Possibilities	NP	(original)
Switching Disadvantages	SD	[54]
Career Disadvantages	CD	[16]
Fear of Competition	FC	(original)
Fear of Data Misuse	FM	(original)
Fear of Losing One’s Unique Value	FV	[71]

We captured researchers’ intentions to share research data with wording adapted from the ‘Intention to Share Knowledge’ scale, which was developed by Bock, Zmud, Kim and Lee [84]. The scale consisted of five items divided into two explicit knowledge items and three implicit knowledge items. One exemplary item from the questionnaire was "I will always provide my manuals, methodologies and models".

To illustrate the perceived value of data exchange, we used the questionnaire developed by Kim and Kankanhalli [85] and adapted its wording to the topic of RDM and data sharing. The questionnaire consisted of three items.

We measured the construct ‘Switching Advantages’ by using the adapted four item scale developed by Kim and Kankanhalli [85], which asked about the benefits of changing current behavior. The questionnaire contained four items, such as "Sharing my primary research data would increase my productivity more than working in the current way", and referred to the improvement of research methods and results due to sharing data.

We measured the construct ‘Career Benefits’ according to a scale developed by Kim and Zhang [16] to record the perceived career advantage of data exchange. We slightly modified this construct by addressing the advantage of raw data exchange instead of general research data exchange.

We measured the construct ‘Switching Disadvantages’ by using the wording-adapted scale of Kim and Zhang [16]. The construct was comprised of four items, such as "It would cost me a lot of resources to share my primary research data", and included both time and organizational effort.

In order to capture which career disadvantages researchers associated with sharing data, we used a questionnaire developed by Kim and Zhang [16], consisting of four items in a minimally modified form.

To measure the extent to which researchers were afraid of losing their own value within research after making their research data available to others, we adapted Renzl’s items [71].

We distinguished performance and career advantages from perceived disadvantages, as they represent individual beliefs for which no corresponding antipoles exist. A researcher who disagrees with the statement that ‘open data provides career advantages’ does not necessarily believe that it offers disadvantages. Therefore, we measured career disadvantages on a different scale. This distinction was adapted from Cenfetelli [86] and Cenfetelli and Schwarz [87], who described the differentiation of enablers and inhibitors on divergent scales. The questionnaire as well as the presented survey data are freely available for reuse. Table 1 provides an overview of all original items and items derived from literature. We measured all items in part two on a 5-point Likert scale (“Strongly Agree”–“Strongly Disagree”).

The relevant descriptive statistics of our study are shown in Table 2.

**Table 2:** Overview of descriptive statistics.

Participants in total	N = 995			
Distribution of affiliations	276 associate / full professors	193 post-docs / lecturers	489 PhD students / candidates	37 part time / student assistants
Distribution of research disciplines	344 humanities researchers	236 engineering researchers	162 life science researchers	253 science researchers

## Results

To test the hypotheses with a structural equation model (SEM), we first conducted an exploratory factor analysis to determine the factor structure for further analysis. Next, we evaluated the measurement model and the SEM by criteria of quality. The structural equation model was then calculated. Finally, we performed the post-hoc analysis.

### Exploratory factor analysis

In order to examine correlative structures in our data set, we conducted an exploratory factor analysis using SPSS version 24.0.0.0. The Promax rotation value was set to kappa = 4, resulting in ten factors with 38 items. We assessed reliability, since the composite reliability (CR) of all constructs was above the threshold of 0.70 [89]. All constructs reached a Cronbach’s Alpha (CA) above the recommended threshold of 0.70 [90]. We assumed convergent validity since the average variance extracted (AVE) for each construct exceeded the threshold of 0.50 [89]. We checked all items for discriminant validity, leading to the exclusion of those with high cross loadings. The measurements are listed in the S1 Appendix. Before testing the hypotheses, we ensured that the measurement was not influenced by common method bias (CMB). To alleviate concerns about CMB, we tested multicollinearity using the variance inflation factor (VIF), resulting in values between 1.00 and 3.00, which is lower than the suggested maximum values of 3.30 [91]. Therefore, the data set was not affected by CMB. The relevant values are listed in the S1 Appendix.

### Structural model

We carried out the standard statistical procedures using R version 3.5 and the lavaan software package. To evaluate our model, we used the structural equation modell (SEM). First, confirmatory factor analysis (CFA) was computed to check the properties of the measurement scales. We modelled all of our hypothesized constructs as reflective measures.

For the evaluation of model fits, we applied standard criteria [92]: the overall model fit was assessed based on a chi^2^ goodness-of-fit test, comparative fit indices (CFI/TLI; values above 0.90 indicate a good fit, values above 0.95 an excellent fit), root mean square error of approximation (RMSEA; values below 0.08 indicate an acceptable fit, values below 0.05 an excellent fit; p-close-value should be above 0.5.), and standardized root mean square residual (SRMR; values below 0.08 indicate good fit with the data). The estimation of our model resulted in good to excellent fit parameters (CFI = 0.961; TLI = 0.955; RMSEA = 0.046 with a 90 percent confidence interval; SRMR = 0.046). The chi^2^ test was significant with chi^2^ = 1,819.18, df = 584, p < 0.001. While the high chi^2^ value can be explained by the large sample size [93], the overall model fits meet the standard requirements for CB-SEM studies [92].

### Measurement model

**Fig 1:**
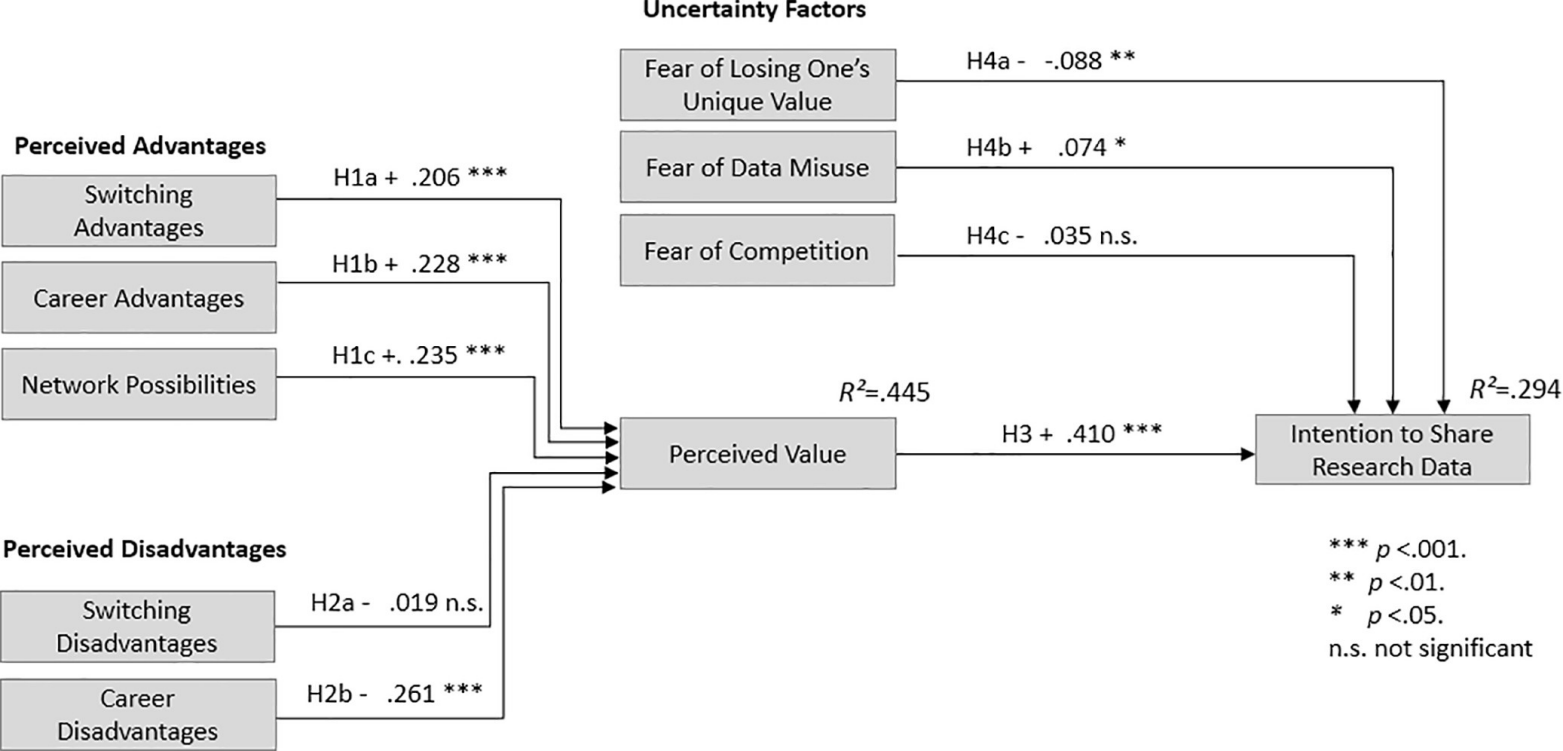
Structural equation model: Modified value-based model of technology adoption.

Next, we calculated the actual SEM, which is visualized in Fig 1.

The proposed theoretical model on latent dimension yielded a good fit with the data, with CFI = 0.957; TLI = 0.952; RMSEA = 0.048 with a 90 percent confidence interval; SRMR = 0.059). The chi^2^ test was significant with chi^2^ = 1,933.71, df = 592, p < 0.001.

Overall, the model we developed explained nearly 30% (R^2^ = 0.294) of the variance of the intention to share research data (see Fig 1).

Additionally, nearly 45% (R^2^ = 0.445) of the variance of the perceived value could be explained by our model (see Fig 1). In Fig 1 we especially visualized the effects of the constructs and the significations of our hypotheses.

Table 3 summarizes the results of our survey and the confirmation of the main hypotheses. Overall, eight out of eleven hypotheses were confirmed. We describe the findings regarding H5 and H6 in the following section in more detail.

**Table 3:** Statistical results of the survey data.

Predictor	Standardized Beta	S.E.	p-value	T value	Hypotheses
PV–SA	0.206	0.035	0.000	5.931	H1a: confirmed
PV–CA	0.228	0.040	0.000	5.688	H1b: confirmed
PV–NP	0.235	0.042	0.000	5.562	H1c: confirmed
PV–SD	-0.019	0.027	0.474	-0.717	H2a: not confirmed
PV–CD	-0.261	0.028	0.000	-9.461	H2b: confirmed
IS–PV	0.410	0.030	0.000	13.448	H3: confirmed
IS–FM	0.074	0.028	0.009	2.628	H4b: not confirmed
IS–FC	0.035	0.030	0.244	1.165	H4c: not confirmed
IS–FV	-0.088	0.031	0.004	-2.889	H4a: confirmed

### Differences between disciplines and hierarchical levels

In order to test the various difference hypotheses that were set up as post-hoc analyses in addition to the structural equation model, we calculated a one-factorial ANOVA using the SPSS 25 statistical program. First, we tested if there was a significant difference between research disciplines in the perception of a career advantage due to sharing data.

The ANOVA calculation showed a significant difference (f = .16, F (3,991) = 8.07, p < .001), confirming H5. Looking at the results of the Tukey HSD Post-Hoc Test, we found a significant difference between humanities and social scientists and engineering scientists (-.28, 95% CI [-51, -.05], p = .008) as well as between natural scientists and engineering scientists (-.46, 95% CI [-.70, -.21], p < .001). Accordingly, the perceived career advantage of both humanities and natural scientists was significantly smaller than that of engineering scientists. Engineering scientists thus perceived the biggest career advantage in sharing data. Regarding the fear that others might misuse open research data, we found that the variance homogeneity was p = .129, and a significant difference (f = .05, F (3,991) = 2.63, p = .049). Thus, humanities scientists differed significantly from life scientists (-.30, 95% CI [-58, -.01], p = .038) in their fear of data misuse. Researchers in the humanities (M = 2.89, SD = 1.16) therefore had a significantly lower fear of data misuse than life scientists (M = 3.19, SD = 1.21). Although the variance homogeneity could be demonstrated (p = .056) with regard to the assumed difference in perceived fear of competitive pressure, we found no significant result in the calculation of the one-factorial ANOVA (f = .08, F (3,991) = 1.71, p = .163). Researchers of various disciplines therefore did not differ in perceived competitive pressure. The assumption that the intention to share data differs between the different disciplines was also correct. With p = .048, there was no variance homogeneity, so we performed an additional Welch’s *t*-test (f = .08, F (3,488.75) = 2.89, p = .035). Subsequently, the Games-Howell test showed significant differences (-.23, 95% CI [-44, -.19], p = 0.26) between researchers in engineering (M = 3.42, SD = .82) and life science researchers (M = 3.65, SD = .79). Life scientists, therefore, had a greater intention to share data than engineering scientists.

Finally, we examined the differences between hierarchical levels. When perceiving the career benefits of sharing data, the variance homogeneity was violated with p < .001. Therefore, we performed a Welch’s *t*-test, which yielded a significant result (f = .13, F (3,157.43) = 5.30, p = .002). In order investigate which groups significantly differ from each other, we used the Games-Howell test. The test showed that professors (-.27, 95% CI [-49, -.06], p = .006) as well as post-docs (-.27, 95% CI [-, -.04], p = .015) differed significantly in their perceptions of career advantages compared to research associates. Research associates perceived the most benefit to their careers in relation to data sharing (M = 3.13, SD = .97), whereas professors (M = 2.86, SD = 1.16) and post-docs (M = 2.86, SD = 1.11) hardly differed in this perception. Thus, H6 was confirmed.

## Discussion

Previous studies have already indicated that individual factors significantly impact researchers’ willingness to share data. However, this work not only identified which factors prevent or promote behavior, but also how these factors are related. Furthermore, we considered how the factors differ between academic disciplines and positions at European research institutions. We could confirm 8 out of our 11 hypotheses, which have yielded interesting conclusions. Some of the findings can be explained with existing research but other aspects will need to be investigated in future research.

The value-based model developed by Kim [20] proved to be suitable for measuring why individuals share their data. Most of the measured constructs had a significant impact on the intention to share data, as did perceived value. It could also be shown that the prospect theory [21] improved our understanding of the process of data exchange. Uncertainties seem to have an impact on the intention to share data (H4a and H4b: confirmed).

The findings showed that career advantages have a positive impact on data sharing (H1b: confirmed). This has also been demonstrated in previous studies. On the one hand, recognition and improvement of one’s status or reputation can be motivators for sharing knowledge [94]. On the other hand, it has been shown that career advantages should be considered and worked out more. For example, Kim and Stanton [19] have shown that researchers who take more advantage of their careers in recognition of their own work and increase academic standing are more willing to share their data with others. Fecher et al. [72] have stated that researchers are no longer using money as an effective driver for increasing data sharing but rather for gaining reputation.

Career Disadvantages had a significant impact on perceived value (H2b: confirmed). This matches the results of Fechter et al. [72], who identified these as potential causes that prevent people from sharing their data. Tenopir et al. [36] labeled them as barriers due to the lack of support for data exchange as well. The factor Switching Costs was not significant (H2a: not confirmed) in the calculation of the structural equation model. This differs from the findings of Kim & Zhang [16].

In addition, a significant influence of uncertainty factors was demonstrated in this study (H4a and H4b: confirmed). It was found that the fear of losing one’s own value by providing the research data had a significant negative impact on the intention to share data (H4a: confirmed). In other studies, this influence was identified as well. For example, researchers’ fears that others would publish their own data first was identified as the most common anxiety that resulted in not making data publicly available [72]. By sharing data, researchers feel that they are sacrificing the opportunity to get multiple publications from a single data set because other researchers may use the data as a source for publications, too [69]. Furthermore, a significant influence of the fear of data misuse on the intention to provide data could be ascertained (H4b: confirmed). Whereas previous studies identified this factor as a barrier [46], this article found a positive influence on the intention to share data. While this may seem contradictory at first, it may be explained in terms of research guidelines. Researchers may perceive that data that has been appropriately processed with metadata will counter data misuse because metadata can provide a clear contextual categorization. While other studies identified competitive pressure as a barrier [18], this could not be confirmed within this study (H4c: not confirmed). This could possibly be due to the fact that the influence of this factor was directly related to the actual sharing of data and not to the intention to share. Likewise, this factor has only been identified as a barrier in qualitative studies so far but has not been tested in theoretical models regarding the decision-making process. Another possible reason for the positive influence of competitive pressure on the intention to share data is that publishing data may increase the visibility and reputation of researchers [14]. Nevertheless, most of these uncertainties are not grounded in rational thought and therefore could be eliminated by raising awareness about the benefits of RDM. Researchers, therefore, need to be sure that there are no negative consequences for them when they share their data.

In previous German research contexts, interdisciplinary differences with regard to data exchange had already been detected [18]. In this study, these differences could be confirmed to a large extent (H5: confirmed). In many international studies, reluctance to share data has been attributed to the increased fear of data misuse [62,95]. However, within this work, we showed that researchers in the humanities and social sciences had the least fear of data abuse. Unlike earlier studies from the US, it was not possible to identify scientists as pioneers in data exchange. Although this study also showed that scientists in Germany have a significantly higher intention and actually share significantly more data than humanitarian scientists, the mean was slightly higher in life science. This fits with the results of Tenopir et al. [36] who found that lack of recognition for work was a major reason that scientists did not share data.

We also identified differences between hierarchical levels in the considered German institutions (H6: confirmed). Consistent with the results of Tenopir et al. [36], we found that younger researchers had a greater intention of sharing data. This may be due to the fact that the careers of young researchers depend on publications [75] and that they may be exposed to co-authorship through the sharing of data. In addition, research associates perceived the lowest cost of labor with respect to sharing data while professors perceived the highest costs. The difference may be attributed to the potential financial compensation associated with providing research data: since research associates have lower incomes than professors, they are more likely to be motivated by such financial incentives.

### Implications

The intention to share research data is a basic prerequisite for the acceptance of e-science technologies, allowing researchers to *conduct research that implements research data sharing and reuse practices (open data)*. However, previous strategies to make these technologies more attractive have primarily focused on the improvement of technical features and characteristics. While this practice-oriented approach is initially comprehensible, the grounds for researchers’ acceptance towards technologies cannot be solely attributed to technical features and improvements. In this regard, our study focused on the non-technical perspective of open data acceptance, and further demonstrated the impact of psychological motivators and barriers. These motivators and barriers were related in a comprehensive model that can be used by researchers and practitioners to understand why researchers do not share their data. By combining the value-based theory [20] and the prospect theory [21], as well as some original constructs, we were able to show which non-technical factors influenced researchers’ willingness to share data and how.

This research has several practical implications for universities, IT executives in higher education, and academic libraries that provide data services for researchers. Even though the model was tested in German academic institutions, other research results in the literature suggest that our results can also be applied to the international context.

Grounded in the value-based theory, this work investigated the relevance of value processing in the context of open data acceptance. Hence, we examined to what extent value constitutes a significant mediator between individuals’ evaluations and their intentions to share research data. Our results confirmed the hypothesis that open data adoption depends on researchers’ evaluation processes (weighing out costs and benefits against one another). This process is significantly determined by beneficial factors such as performance advantages and career advantages, as well as hindering factors such as career disadvantages. Although previous research had assumed that researchers expect a benefit for the community rather than for themselves in data exchange, our results indicate that researchers–especially in Europe–are indeed aware of personal benefits, including career advantages as well as performance advantages. Furthermore, the results showed that individual disadvantages do not outweigh individual advantages, which constitutes an interesting finding in the context of open data. The degree to which personal benefits and benefits for the community arise varies in relation to disciplines and hierarchy levels (e.g. professors, post-docs and PhD students). Moreover, researchers were not negatively affected by the additional effort of data preparation and release, which is an unexpected result that deviates from the results of prior studies in which researchers concluded that efforts involved in the data sharing process hinder that process [52,53].

Our results also show that several uncertainty factors influence researchers’ decision-making processes–with respect to open data–to different extents. While the fear of misuse had no significant impact on researchers’ intentions to embrace open data, the positive impact of fear of competition has been of great interest to other researchers who identified it as a negative impact factor [18,57]. The non-significant impact of the fear of misuse could be explained by the fact that accessible research data has already been worked with and published; misuse of such data by third parties (i.e. other researchers) could lead to serious consequences. Additionally, the visibility and accessibility of data in suitable repositories can serve to anticipate publications and disclose results before being subjected to long-term review processes. In this case, the publication of data records can lead to competitive advantages. While the positive influence of fear of competition seems counterintuitive, it is explained by the observations of Piwowar and Vision [14], who stated that RDM and open data increase the visibility and reputation of researchers in the community. This seems to lead to a competitive advantage from researchers’ perspectives.

## Conclusions

In this article, we examined researchers’ willingness to share data in relation to individual factors based on the value-based model of technology acceptance and influenced by the prospect theory. We showed how uncertainty factors and perceived values influence researchers’ intentions to adopt the open data process. We found that the prospect theory developed by Kahneman and Tversky [21] was well suited to our modified value-based model of technology acceptance. To derive our model, we used PLS SEM and tested the relations of individual constructs. We consulted 995 researchers from 13 large to medium-sized universities in Germany, which constitutes the largest known sample in the German research context at this time. We confirmed eight of our eleven hypotheses.

Out of all the individual factors that were postulated to affect the sharing of data, we identified five that had a significant impact. In particular, perceived value had a major impact on the intention to share research data. Furthermore, we found that personal uncertainties still influence the intention to share data, even if clear advantages regarding the data exchange have already been acknowledged. We found that the fear of losing perceived value by providing the research data had a significant negative impact on the intention to share data. In addition, we found differences between hierarchical levels and disciplines that highlight the diversity of perceived values, uncertainties and intentions in the research area.

We focused on European academia because of an apparent gap in the research literature on the subject of open data.

## Limitations & outlook

The presented findings include diverse limitations that should be addressed in future research. The survey focused on the general intention to share research data, ignoring domain-specific regulations and policies: researchers may be prevented from sharing research data due to ethical or legal aspects. To control such domain-specific peculiarities, descriptive data need to be taken into consideration. While previous studies on descriptive open data acceptance focused on researchers in different countries (e.g. Netherlands, Austria, US), the participants of this study were researchers from nine German universities that have already started initiatives to educate researchers about open data practices. It can be assumed that the participants had already accrued some experience with open data and therefore were informed about the details of data exchange. This might explain why the value of open data is more likely to be uncovered by positive determinants and why uncertainty factors only partly indicate unexpected effects. Further research could compare these findings with data from other German universities. A second aim for future investigations might be to examine a possible moderation of different research disciplines regarding the impact of uncertainty factors. Bauer et al. [18] have stated that the fear of competition was strongly indicated by researchers in biology, while other disciplines were not affected as much. This claim is supported by Wilms et al. [12] who have stated that disciplinary differences indeed exist. Although these findings help us to understand the impact of fear of competition, we lack an explanation as to why it constitutes a driver instead of a hindering factor. Additionally, further research should examine hierarchical levels: differences between the professorship level and the academic mid-level (i.e. academic members and PhD candidates) can indicate whether future implementation strategies should be directed in a top-down fashion or introduced by the next generation of academics.

This work constitutes a basis upon which future research can be carried out, and we hope that it will motivate scholars to explore interesting avenues in the field of open data.

## Supporting information

S1 Appendix (DOCX)
