# Supplementary material for: When are researchers willing to share their data? – Impacts of values and uncertainty on open data in academia
Source: PLoS One. 2020 Jul 1;15(7):e0234172. doi: 10.1371/journal.pone.0234172 (PMC7329060; doi:10.1371/journal.pone.0234172)
Supplement: S1 Appendix — (DOCX) [file pone.0234172.s001.docx]

**Appendix**

**Table 2.** Factor charges of the exploratory factor analysis using the principal component analysis with Varimax rotation

|  | 1 | 2 | 3 | 4 | 5 | 6 | 7 | 8 | 9 | 10 |
| --- | --- | --- | --- | --- | --- | --- | --- | --- | --- | --- |
| IS_01 | **0,448** | 0,12 | 0,089 | -0,005 | -0,018 | 0,039 | -0,172 | 0,209 | 0,099 | -0,314 |
| IS_02 | **0,839** | 0,077 | -0,043 | 0,084 | -0,101 | 0,004 | 0,003 | -0,073 | -0,064 | 0,209 |
| IS_03 | **0,797** | -0,144 | 0,1 | 0 | -0,003 | -0,021 | 0,064 | 0,008 | 0,052 | -0,229 |
| IS_04 | **0,861** | 0,084 | -0,141 | 0,006 | 0,069 | 0,017 | 0,03 | -0,108 | -0,152 | 0,329 |
| IS_05 | **0,757** | -0,064 | 0,04 | -0,087 | 0,069 | -0,028 | 0,024 | 0,072 | 0,095 | -0,209 |
| PV_1 | 0,024 | **0,879** | -0,024 | 0,008 | 0,028 | -0,062 | 0,041 | -0,009 | 0,027 | -0,045 |
| PV_2 | 0,036 | **0,893** | 0,013 | 0,013 | -0,008 | 0,068 | -0,039 | -0,034 | -0,007 | 0,027 |
| PV_3 | -0,042 | **0,906** | 0,05 | -0,035 | -0,007 | -0,012 | 0,045 | 0,018 | 0,028 | -0,078 |
| SA_01 | -0,01 | 0,038 | **0,884** | 0,007 | 0,045 | 0,022 | 0,013 | -0,062 | 0,004 | 0,021 |
| SA_02 | -0,015 | 0,001 | **0,978** | -0,007 | -0,04 | -0,005 | 0,04 | -0,031 | -0,033 | 0,046 |
| SA_03 | -0,022 | 0,026 | **0,956** | 0,005 | -0,051 | -0,031 | 0,04 | 0,024 | -0,043 | 0,038 |
| SA_04 | 0,009 | -0,031 | **0,801** | 0,015 | 0,112 | 0,029 | -0,109 | -0,004 | 0,015 | 0,027 |
| CA_01 | 0,015 | -0,033 | -0,043 | **0,865** | 0,068 | -0,017 | -0,002 | 0,043 | -0,042 | -0,019 |
| CA_02 | -0,021 | 0,005 | -0,03 | **0,913** | 0,057 | 0,014 | -0,006 | -0,011 | 0,045 | -0,056 |
| CA_03 | 0,004 | 0,001 | -0,002 | **0,878** | 0,066 | 0,008 | -0,011 | -0,002 | 0,052 | -0,037 |
| CA_04 | 0,025 | 0,01 | 0,106 | **0,859** | -0,04 | -0,006 | -0,015 | 0,001 | -0,03 | -0,02 |
| NP_01 | 0,037 | 0,01 | 0,029 | -0,009 | **0,884** | -0,002 | 0,017 | -0,008 | -0,005 | 0,011 |
| NP_02 | -0,016 | -0,014 | 0,009 | 0,004 | **0,925** | 0,005 | 0,066 | -0,029 | -0,011 | -0,069 |
| NP_03 | -0,049 | 0,026 | 0 | 0,004 | **0,901** | 0,003 | 0,097 | 0,029 | -0,03 | -0,07 |
| NP_04 | -0,033 | 0,021 | -0,015 | 0,008 | **0,921** | -0,004 | -0,048 | 0,047 | -0,023 | 0,025 |
| NP_05 | 0,064 | -0,04 | 0,007 | 0,064 | **0,813** | 0,002 | -0,119 | -0,067 | 0,071 | 0,148 |
| SD_01 | -0,007 | 0,004 | -0,03 | 0,016 | 0,019 | **0,903** | -0,05 | 0,009 | 0,048 | -0,072 |
| SD_02 | -0,032 | 0,042 | -0,069 | 0,003 | 0,055 | **0,928** | 0,032 | 0,041 | -0,036 | -0,065 |
| SD_03 | -0,011 | 0,032 | 0,053 | -0,017 | -0,022 | **0,875** | -0,025 | 0,007 | 0,01 | 0,053 |
| SD_04 | 0,046 | -0,098 | 0,064 | -0,003 | -0,056 | **0,75** | 0,057 | -0,04 | -0,037 | 0,121 |
| CD_01 | 0,006 | 0,046 | -0,011 | -0,009 | 0,008 | 0,005 | **0,931** | 0,056 | -0,027 | -0,051 |
| CD_02 | 0,039 | 0,013 | 0,01 | -0,031 | 0,031 | -0,041 | **0,848** | 0,081 | 0,078 | -0,034 |
| CD_03 | 0,031 | -0,023 | -0,01 | 0,021 | -0,004 | 0,031 | **0,489** | -0,182 | 0,541 | -0,028 |
| CD_04 | 0,005 | -0,048 | 0,009 | 0,006 | -0,059 | 0,075 | **0,664** | -0,024 | 0,286 | -0,012 |
| FC_01 | -0,023 | -0,013 | -0,023 | -0,002 | -0,044 | 0,023 | 0,052 | **0,896** | 0,021 | 0,079 |
| FC_02 | -0,027 | -0,007 | -0,046 | 0,028 | 0,022 | 0,001 | 0,06 | **0,897** | -0,005 | 0,096 |
| FM_01 | 0 | -0,023 | -0,028 | 0,004 | 0,024 | -0,021 | 0,004 | 0,022 | **0,878** | 0,083 |
| FM_02 | -0,008 | 0,025 | -0,013 | 0,035 | -0,046 | -0,012 | -0,024 | -0,002 | **0,946** | 0,064 |
| FM_04 | -0,034 | 0,029 | -0,008 | -0,004 | 0,009 | 0,001 | -0,072 | 0,007 | **0,969** | 0,06 |
| FM_05 | -0,031 | 0,032 | -0,02 | -0,021 | 0,022 | -0,003 | -0,059 | 0,004 | **0,943** | 0,042 |
| FV_02 | 0,089 | -0,078 | 0,06 | -0,113 | 0,001 | 0,034 | -0,147 | 0,08 | 0,195 | **0,798** |
| FV_03 | 0,007 | 0,028 | 0,107 | 0,056 | 0,042 | -0,052 | 0,248 | 0,231 | 0,113 | **0,561** |

**Table 3.** Results of the exploratory factor analysis

| **Factor** | **Items** | **M** | **SD** | **α** | **AVE** | **CR** |
| --- | --- | --- | --- | --- | --- | --- |
| Intention to share data | 4 | 3.53 | .81 | .83 | .51 | .82 |
| Perceived Value | 3 | 3.19 | .98 | .89 | .74 | .89 |
| Switching Advantages | 4 | 2.50 | .96 | .92 | .76 | .92 |
| Career Advantages | 4 | 3.00 | 1.06 | .94 | .80 | .94 |
| Network Possibilities | 5 | 3.42 | 1.01 | .94 | .76 | .94 |
| Sharing Disadvantages | 4 | 3.27 | 1.00 | .89 | .68 | .88 |
| Career Disadvantages | 4 | 2.94 | 1.08 | .89 | 68 | .90 |
| Fear of having to compete | 2 | 2.57 | 1.11 | .92 | .85 | .92 |
| Fear of Misuse | 4 | 2.98 | 1.17 | .95 | .83 | .95 |
| Fear of losing unique’s value | 2 | 2.47 | 1.14 | .88 | .78 | .88 |

**Table 4.** Table illustrating the discriminant validity (The thick printed values are the square roots of the AVE values, the other values are the correlations of the constructs)

| **Factor** | 1 | 2 | 3 |  | 4 | 5 | 6 | 7 | 8 | 9 | 10 |  |
| --- | --- | --- | --- | --- | --- | --- | --- | --- | --- | --- | --- | --- |
| 1: SI | **.71** |  |  |  |  |  |  |  |  |  |  | |
| 2: PV | .50 | **.86** |  |  |  |  |  |  |  |  |  | |
| 3: SB | .41 | .42 | **.87** |  |  |  |  |  |  |  |  | |
| 4: CA | .44 | .53 | .47 |  | **.90** |  |  |  |  |  |  | |
| 5: NP | .52 | .53 | .50 |  | .73 | **.87** |  |  |  |  |  | |
| 6: SC | .02 | -.09 | -.04 |  | 0.04 | .04 | **.82** |  |  |  |  | |
| 7: CD | -.11 | -.35 | -.00 |  | -.11 | -.10 | ..28 | **.83** |  |  |  | |
| 8: FC | -.04 | -.14 | .13 |  | .12 | .06 | .27 | .63 | **.92** |  |  | |
| 9: FM | -.08 | -.27 | -.03 |  | -.07 | -.08 | .30 | .77 | .60 | **.92** |  | |
| 10: FV | -.17 | -.25 | .06 |  | -.06 | -.09 | .18 | .70 | .60 | .61 | **.88** | |
